# Treatment Outcomes of Dolutegravir- Versus Efavirenz-Based Highly Active Antiretroviral Therapy Regimens Among Treatment-Naive People Living With HIV

**DOI:** 10.7759/cureus.40139

**Published:** 2023-06-08

**Authors:** Prarthana R Mahale, Brijalkumar S Patel, Navsin Kasmani

**Affiliations:** 1 College of Medicine, Gujarat Medical Education and Research Society (GMERS) Medical College and Hospital, Valsad, IND; 2 Department of Pharmacology, Gujarat Medical Education and Research Society (GMERS) Medical College and Hospital, Navsari, IND; 3 Department of Medicine, Gujarat Medical Education and Research Society (GMERS) Medical College and Hospital, Valsad, IND

**Keywords:** hiv transmission, naïve hiv-infected patients, highly active antiretroviral treatment, cd4+ cell count, viral load, antiretroviral therapy, efavirenz, dolutegravir

## Abstract

Background: In India, following the implementation of the dolutegravir (DTG) based regimen, only a few studies compared the outcomes of DTG and efavirenz (EFV) based regimens. Therefore, this study aimed to assess virological suppression and gain in CD4+ counts of DTG and EFV-based antiretroviral therapy (ART) regimens.

Methods: A retrospective study was conducted and the entire sample (n=140) was divided into two major classes as DTG group (n=70) and EFV group (n=70) further classified as tenofovir/lamivudine/dolutegravir (TLD) and tenofovir/lamivudine/efavirenz (TLE) regimen. Data was collected on socio-demographic characteristics, laboratory measures, and clinical and drug-related variables. For quantitative and qualitative data analysis, respectively, the T-tests and Chi-square tests were applied.

Results: The mean CD4+ gain was comparable in both regimens after six months of ART but significant after 12 months of ART in the TLD group. Viral load suppression was achieved in 55.71% of clients in the TLE group after six months of ART while in the TLD group, 88.57% of clients achieved virologic suppression which was highly significant. Clients who remained on the DTG-based regimen gained significantly more weight at 12 months (mean +6.15 kg) as compared to the EFV-based regimen (mean +1.85 kg). After 12 months of ART, the majority of laboratory variables were unaffected by either regimen with the exception of serum creatinine and random blood sugar (RBS) in the TLD group.

Conclusions: Our study provides real-life evidence of better outcomes of therapy with DTG over EFV in terms of viral load suppression but immunologic recovery is equivalent in EFV-based regimens after six months of treatment. We recommend the use of DTG only in clients with a high baseline viral load as it costs approximately twice as much as EFV when cost-effectiveness is taken into account.

## Introduction

Human immunodeficiency virus/acquired immunodeficiency syndrome (HIV/AIDS) is a public health problem that has claimed more than 38 million life until 2021 globally [1]. According to the latest statistics, there are roughly 2.4 million people living with HIV (PLHIV) in India [2]. For treatment-naive people with HIV infection, highly active antiretroviral therapy (HAART) is the gold standard of care because it enables long-term virological suppression [3]. Until mid-2018, the World Health Organization (WHO) recommended antiretroviral therapy (ART) regimen for HIV-1 infection comprised a combination of two nucleoside reverse-transcriptase inhibitors (NRTIs) combined with a non-nucleoside reverse-transcriptase inhibitor (NNRTI), namely efavirenz (EFV) at a dose of 600 mg daily (known as EFV 600 study) [4,5]. The EFV-based regimen was then challenged by the landmark Study ING11446 (SINGLE trial), which showed that the dolutegravir (DTG) based regimen had a more sustained viral suppression and immunological recovery than the EFV-based regimen. Recent systematic reviews and meta-analyses showed that DTG has a higher genetic barrier to drug resistance and a lower potential for drug-drug interactions than EFV [6].

By 2019, the WHO recommended transitioning to DTG-based first-line regimens as 12 out of 18 countries surveyed by the WHO reported pre-treatment drug resistance to EFV exceeding the recommended threshold of 10% [7]. In settings with a high prevalence of resistance to NNRTIs, DTG might be a preferred first-line option to EFV. In India, following the implementation of the DTG-based regimen, only a few studies compared the outcomes of DTG and EFV-based regimens in terms of virological suppression and immunological recovery. Real-world studies are therefore required to evaluate the outcome of these new antiretroviral medications in order to validate the use of DTG-based regimens in population treatment programs.

Two primary goals of anti-HIV therapy are to suppress viral HIV RNA plasma replication and preserve and restore the number of circulating CD4+ T cells, the immune cells attacked by HIV. Therefore, this study is aimed to assess virological suppression and gain in CD4+ counts of DTG and EFV-based ART regimens to better understand the long-term outcomes of these combination treatment regimens.

## Materials and methods

A retrospective hospital-based study was carried out at an ART center of a tertiary care hospital. Approval of the research project was obtained from Institutional Human Ethics Committee (No. MCV/IHEC/381). Additionally, approval has been obtained from the respective state AIDS Control Society. The confidentiality of reviewed patient information was maintained throughout the coding of individual patient data.

Study population and study duration

The study was conducted on adult people living with HIV (PLHIV) who were on DTG and EFV-based ART regimens between October 2018 and September 2021 that fulfilled the inclusion criteria.

A total of 140 client files (n=140) were eligible and reviewed. The entire sample was divided into two major classes as DTG group and the EFV group which was further classified as tenofovir/lamivudine/dolutegravir (TLD) and tenofovir/lamivudine/efavirenz (TLE) regimen. A ratio of 1:1 was applied whereby 70 clients on TLD (n=70) and 70 clients on TLE (n=70) were recruited by a simple convenient sampling method. The study was conducted for two months whereby information was extracted using a case record form (CRF).

HIV-positive people who were substituted for DTG-based ART regimen from EFV-based regimen were also noted and the reasons for substitution were evaluated. Additionally, the number of clients who switched to a second-line regimen from DTG and EFV-based first-line regimens was noted.

Selection criteria of people living with HIV

All treatment-naïve adult HIV-positive people (age >18 years) of either sex on DTG and EFV-based first-line regimens for at least six months, who had CD4+ counts done at baseline, six months and at one year (if done) and viral load at six months and at one year (if done), whose records were legible and complete were included in the study. Those that were transferred out/died within less than six months of follow-up and pregnant women were excluded from the study.

Outcome variables

The primary outcome was the proportion of HIV-positive people with a mean increase in CD4+ cell count from baseline to six months and one year. Virological suppression at six months and a year was the secondary endpoint.

Data collection

The CRF consisting of three parts was used in data collection. It was validated before the commencement of the study. The first part of CRF included demographic information such as sex, age, gender, weight, education, marital status, and past/personal and medical history. The second part of CRF included laboratory data, and the third part consisted of details of antiretroviral treatment which included information regarding the type of regimen initiated, the substitution of regimen (within first-line ART), switching of regimen to second-line ART, WHO clinical stage, functional status (W/A/B), CD4+ cell count, viral load, side effects, prophylaxis, and adherence rate (assessed as pill count).

Statistical analysis

Data from the CRF was entered in the Excel sheet. A descriptive statistical study was performed and results were presented by text, tables, and charts. For quantitative and qualitative data, respectively, the T-test (paired and unpaired) and Chi-square tests were applied. The results were considered having statistical significance when P <0.05 and highly significant when P < 0.001.

## Results

In our study, the mean+/-SD age of the study population was 37.56+/-11 and most of the population was within the age group of 34-45 years. The majority of the people living with HIV (PLHIV) were male (93%). Seventy percent of the PLHIV were married, and 51% had HIV-positive spouses. Only 12% of HIV-positive people were homosexual, with 76% being heterosexual, 24.29% of PLHIV had only completed primary school, and 22.14% of PLHIV were illiterate. Tuberculosis was detected in 25% of PLHIV. Alcohol, smoking, and tobacco chewing history were present in 13.57%, 9.29%, and 10.71% of PLHIV, respectively, but this information was not readily available in the majority of client files. The results are presented in Table 1.

**Table 1:** Socio-demographic characteristics of the study population

Patient characteristics		n=140 (%)
Age	Mean+/-SD	37.56+/- 11
	18-34	55 (39.29)
	34-45	59 (42.14)
	>45	26 (18.57)
Sex	Male	93 (66.43)
	Female	47 (33.57)
Marital status	Married	98 (70)
	Single	18 (12.86)
	Divorcee	4 (2.86)
	Widowed	18 (12.86)
	Live-in	2 (1.42)
HIV positive in spouse (if married)		51 (36.43)
Occupation	Service (govt./pvt.)	43 (30.72)
	Housewife	26 (18.58)
	Self-employed/small/large business	21 (15)
	Others	50 (35.7)
Risk factor	Heterosexual	107 (76.43)
	MSM (male-to-male sex)	17 (12.15)
	Others	16 (11.42)
Education	Illiterate	31 (22.14)
	Primary	34 (24.29)
	Secondary	65 (46.43)
	College and above	10 (7.14)
Tuberculosis	Yes	35 (25)
	No	105 (75)
Alcohol	Yes	19 (13.57)
	No	24 (17.14)
	Unknown	97 (69.29)
Smoking	Yes	13 (9.29)
	No	30 (21.42)
	Unknown	97 (69.29)
Tobacco	Yes	15 (10.71)
	No	28 (20)
	Unknown	97 (69.29)

According to the NACO recommendations, most HIV-positive people who were receiving EFV had switched to a regimen based on DTG by July 2020. There were no clients switched from the first to the second line of treatment in either regimen. The majority of the clients in our study were in a working-functional state (95.71% at baseline and 98.57% at 12 months) for both regimens.

As shown in Table 2, the mean (+/-SD) CD4+ counts gained in six months from the baseline in the TLE group was 141.086 (+/-10.83), and in the TLD group was 147.514 (+/-56.70). The gain was slightly more in the TLD-based regimen which is statistically non-significant. In 12 months, the mean (+/-SD) CD4+ count gained from the baseline in the TLE group was 183.371 (+/-54.87) while in the TLD group, it was 217.171 (+/-77.17). Here, the mean CD4+ counts gain was more observed in the TLD-based regimen as compared to the TLE-based regimen which is also statistically significant.

**Table 2:** Mean CD4+ changes from baseline to six months and 12 months P value<0.001 is considered statistically significant. TLE: tenofovir/lamivudine/efavirenz; TLD: tenofovir/lamivudine/dolutegravir

Regimen	Baseline	6 months	12 months	Gain 0-6 months		Gain 0-12 months		Gain 6-12 months	
	Mean (+/-SD)	Mean (+/-SD)	Mean (+/-SD)	Mean (+/-SD)	P value	Mean (+/-SD)	P value	Mean (+/-SD)	P value
TLE (n =70)	354.914 (255.10)	496 (265.93)	538.286 (309.97)	141.086 (10.83)	< 0.001	183.371 (54.87)	< 0.001	42.286 (44.05)	0.0035
TLD (n =70)	229.671 (176.69)	377.186 (233.39)	446.843 (253.87)	147.514 (56.70)	< 0.001	217.171 (77.18)	< 0.001	69.657 (20.48)	< 0.001
Mean CD4+ gain difference between both regimens; P value				0.3531		0.0033		< 0.0001	

Viral load suppression was achieved in 55.71 % of PLHIV in the TLE group after six months of ART while in the TLD group, 88.57% of PLHIV achieved virologic suppression which was highly significant. The findings were consistent at 12 months and 95.71% of PLHIV had achieved viral load suppression as compared to 80% in the TLE group (Table 3).

**Table 3:** Viral load suppression in both regimens A viral load <150 copies/mL is considered virological suppression. P value<0.001 is considered statistically significant. TLE: tenofovir/lamivudine/efavirenz; TLD: tenofovir/lamivudine/dolutegravir

Regimen	Viral load suppression (<150 copies/mL)	
	At 6 months N (%)	At 12 months N (%)
TLE (n=70)	39 (55.71)	56 (80)
TLD (n=70)	62 (88.57)	67 (95.71)
P value	< 0.0001	< 0.0044

**Figure 1:**
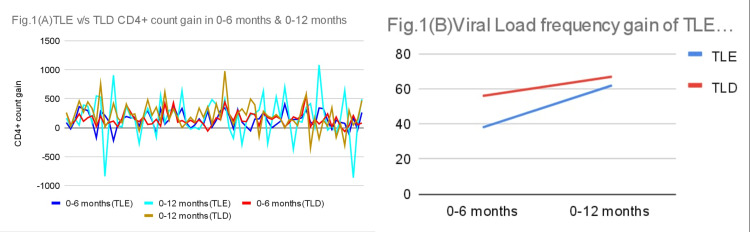
(A) CD4+ gain difference between both regimens, (B) Viral load frequency gain TLE: tenofovir/lamivudine/efavirenz; TLD: tenofovir/lamivudine/dolutegravir

Figure 1A shows that the increase in CD4+ count was more in TLD as compared to TLE over the period of 12 months. There is a higher frequency of PLHIV who have gained viral load suppression in the TLD regimen as compared to TLE during (0-6) and (0-12) months (Figure 1B).

People living with HIV who remained on a DTG-based regimen gained significantly more weight at 12 months (mean +6.154 kg) as compared to an EFV-based regimen (mean +1.857 kg) and the difference was statistically significant. The rate of weight gain slowed in the TLE group from 6-12 months of ART treatment. The results are presented in Table 4.

**Table 4:** Changes in weight gain (kg) from baseline TLE: tenofovir/lamivudine/efavirenz; TLD: tenofovir/lamivudine/dolutegravir

Regimen	Baseline	6 months	12 months	Gain 0-6 months		Gain 0-12 months		Gain 6-12 months	
	Mean(+/-SD)	Mean(+/-SD)	Mean(+/-SD)	Mean(+/-SD)	P value	Mean(+/-SD)	P value	Mean(+/-SD)	P value
TLE (n=70)	53.231 (11.15)	55.187 (10.91)	55.087 (11.45)	1.956 (0.24)	< 0.001	1.857 (0.30)	< 0.001	-0.097 (0.53)	0.6183
TLD (n=70)	49.954 (13.39)	53.809 (12.32)	56.109 (12.37)	3.854 (1.07)	< 0.001	6.154 (1.03)	< 0.001	2.300 (0.05)	<0.00001
Weight gain comparison between both regimens: P value				< 0.0001		< 0.0001		< 0.0001	

The majority of laboratory parameters were unaffected by either regimen after 12 months of treatment. But after 12 months of treatment, serum creatinine value in the TLD group increased from 0.89 to 1.08 (normal range: 0.7-1.2mg/dL), which is likewise statistically significant. Similarly, random blood sugar (RBS) was also affected in TLD-treated PLHIV though it was within the normal range (Table 5).

**Table 5:** Effect on laboratory parameters TLE: tenofovir/lamivudine/efavirenz; TLD: tenofovir/lamivudine/dolutegravir; Hb: hemoglobin; SGOT: serum glutamic-oxaloacetic transaminase; SGPT: serum glutamic pyruvic transaminase

TLE Regimen				
Parameter	Baseline (Mean +/- SD)	12 months (Mean +/- SD)	Gain at 0-12 months (Mean +/- SD)	P value
Hb (g/dL)	12.014 (2.24)	13.149 (2.74)	1.134 (1.00)	-
Serum creatinine (mg/dL)	0.786 (0.19)	0.837 (0.20)	0.051 (0.02)	0.0623
SGOT (units/L)	34.354 (20.15)	32.871 (13.45)	(-1.483) (6.70)	0.7154
SGPT (units/L)	31.253 (24.65)	30.757 (14.37)	(-0.496) (10.28)	0.5557
Random Blood sugar (RBS) (mg/dL)	100.559 (29.42)	96.329 (27.28)	(-4.23) (2.13)	0.8149
TLD regimen				
Hb (g/dL)	11.143 (2.47)	12.831 (2.10)	1.689 (0.36)	-
Serum Creatinine (mg/dL)	0.887 (0.22)	1.077 (0.29)	0.190 (0.07)	< 0.0001
SGOT (units/L)	38.146 (24.88)	37.314 (19.62)	(-0.832) (5.26)	0.5911
SGPT (units/L)	39.373 (37.02)	38.014 (27.90)	(-1.358) (9.12)	0.5961
Random Blood sugar (RBS) (mg/dL)	91.471 (26.03)	102.143 (40.97)	10.671 (14.94)	0.0013

## Discussion

In our study, a significant increase in mean CD4+ count gain was observed after 12 months of treatment in a TLD-based regimen than a TLE-based regimen. This result is in harmony with a meta-analysis done by Sonya et al. who reported that treatment with DTG resulted in a significantly greater increase in mean CD4+ cell count from baseline at week 48 than EFV [8,9]. WHO conducted a meta-analysis that demonstrated moderately strong evidence of enhanced CD4+ cell counts (mean difference=22.87, 95% Crl: 8.29, 37.40) for DTG relative to EFV [10].

However, we observed that the mean CD4+ counts gained after six months in the TLE group were comparable to the TLD group. Our results suggest that although DTG produced a significantly greater increase in CD4+ T cell count than EFV, additional studies are necessary to better understand the impact of these findings. The increase in mean CD4+ count was rapid during the first six months of treatment in both regimens followed by a slight gain as people living with HIV (PLHIV) were stabilized with the therapy. A similar trend was observed in a study done in Tanzania [11].

We found that virological suppression was significantly higher in DTG-based regimens as compared to EFV-based regimens which are supported by previous studies [4,12]. Rutherford et al. reported that DTG-containing regimens were superior to EFV-containing regimens at 48 weeks and at 96 weeks (RR=1.10, 95% CI 1.04-1.16 and RR=1.12, 95% CI=1.04-1.21 respectively) in terms of viral load suppression [13]. Another study done by Mariana et al. observed that viral suppression by 12 months was 84% with TLE and 90.5% with TLD [4]. We discovered that DTG is superior to EFV, and raising the level of virological suppression is the first step toward enhancing the health of PLHIV.

We observed that DTG based regimen gained significantly more weight compared to EFV based regimen across all time periods. Similar findings were observed in a study done in Tennessee, US in which DTG gained 6.0 kg weight compared to 2.6 kg for NNRTIs at 12 months (P< 0.05) [14]. Similar results were reported from other studies [15,16]. Initiation of ART for treatment-naïve PLHIV is often associated with a short period of weight gain. It was thought to be due to a reduction in basal metabolic rate following the suppression of viral replication and controlling inflammation. Though weight gain after starting ART is common and beneficial to PLHIV, substantial weight gain may increase cardiovascular and metabolic complications [16]. So further studies are needed to confirm these findings in large, multicenter cohorts and investigate the effects of weight gain on lipid markers.

We found that most of the laboratory parameters were not altered significantly in both ART regimens. But the rise in serum creatinine in the TLD-based ART regimen raised concern. Lianfeng Lu et al. found that DTG containing regimen presented with higher serum creatinine elevation and decreased eGFRcr [17]. According to Koteff et al., the administration of DTG lowers the serum creatinine clearance rate (CLcr) in healthy individuals by 10-14% [18]. So kidney function should be monitored more closely in combination with other renal biomarkers in DTG-treated PLHIV and future studies are required in the Indian population. Surprisingly, blood sugar was also elevated with a DTG-based regimen. In a study done by Jamison et al., mean HbA1c in EFV-based regimen fell from 6.4 to 6.0% over 18 months but increased from 6.4 to 6.9% in DTG-based regimen, although changes were not statistically significant [16]. Therefore, further research is necessary into the change in blood sugar levels in DTG-treated individuals.

Limitations of the study

This study was based on secondary data (data extracted from case record files) and information on adverse drug reactions and height was not available. We could not evaluate opportunistic infections as clients were directly transferred to the relevant departments depending upon the infection. P value for hemoglobin could not be assessed as prophylactic modalities, counseling, and blood transfusion (if required) was provided in case of low Hb (<9 gm/dL). It would be better if the prospective, multicentric study could be conducted in the future to confirm our findings.

## Conclusions

Our study provides real-life evidence of the superiority of DTG over EFV in terms of virological suppression in treatment-naïve PLHIV. These results further support the current WHO recommendations to initiate ART with a DTG-based regimen. According to our findings, immunologic recovery is equivalent in EFV-based regimens after six months of therapy, despite the fact that DTG produced a significantly greater increase in CD4+ count after 12 months of ART. We recommend the use of DTG only in HIV-positive with high baseline viral loads as it costs approximately twice as much as EFV when cost-effectiveness is taken into account. The change in blood glucose, serum creatinine level, and weight gain in a DTG-based regimen need to be explored further. Additional studies on a larger scale are needed to better comprehend and validate our findings.
